# *Jabuticaba*-Induced Endothelium-Independent Vasodilating Effect on Isolated Arteries

**DOI:** 10.5935/abc.20160118

**Published:** 2016-09

**Authors:** Daniela Medeiros Lobo de Andrade, Leonardo Luis Borges, Ieda Maria Sapateiro Torres, Edemilson Cardoso da Conceição, Matheus Lavorenti Rocha

**Affiliations:** 1 Laboratório de Farmacologia Cardiovascular - Faculdade de Farmácia - Universidade Federal de Goiás (UFG), Goiânia, GO - Brazil; 2 Laboratório de Pesquisa em Produtos Naturais - Faculdade de Farmácia - Universidade Federal de Goiás (UFG), Goiânia, GO - Brazil

**Keywords:** *Jabuticaba* (Myrciaria Cauliflora), Trees, Vasodilatation, Calcium Channels, Muscle, Smooth Vascular

## Abstract

**Background:**

Despite the important biological effects of jabuticaba, its actions on the cardiovascular system have not been clarified.

**Objectives:**

To determine the effects of jabuticaba hydroalcoholic extract (JHE) on vascular smooth muscle (VSM) of isolated arteries.

**Methods:**

Endothelium-denuded aortic rings of rats were mounted in isolated organ bath to record isometric tension. The relaxant effect of JHE and the influence of K^+^ channels and Ca^2+^ intra- and extracellular sources on JHE-stimulated response were assessed.

**Results:**

Arteries pre-contracted with phenylephrine showed concentration-dependent relaxation (0.380 to 1.92 mg/mL). Treatment with K^+^ channel blockers (tetraethyl-ammonium, glibenclamide, 4-aminopyridine) hindered relaxation due to JHE. In addition, phenylephrine-stimulated contraction was hindered by previous treatment with JHE. Inhibition of sarcoplasmic reticulum Ca^2+^ ATPase did not change relaxation due to JHE. In addition, JHE inhibited the contraction caused by Ca^2+^ influx stimulated by phenylephrine and KCl (75 mM).

**Conclusion:**

JHE induces endothelium-independent vasodilation. Activation of K^+^ channels and inhibition of Ca^2+^ influx through the membrane are involved in the JHE relaxant effect.

## Introduction

Cardiovascular diseases are a major cause of death worldwide, among which hypertension accounts for 9.4 million deaths per year.^1^ Around 1 billion adults in the world have hypertension, and that figure will have increased by 25% in 10 years.^2^

Vascular tonus regulation is fundamental to appropriate blood pressure control. Blood vessel contraction and dilation in response to physiological demands are controlled by changes in the intracellular concentration of Ca^2+^ in vascular smooth muscle (VSM) cells. The increase in intracellular concentration of Ca^2+^ occurs via both Ca^2+^ influx through the plasma membrane and Ca^2+^ release from inner sources, such as the sarcoplasmic reticulum.^3,4^ Effective drugs to blood pressure control, such as nifedipine, verapamil and diltiazem, which act as Ca^2+^ channel blockers, induce vasodilation and reduce blood pressure.^5^

The use of natural products as an alternative treatment for hypertension has been extensively studied, being known to induce hypotension with minimum side effects.^6,7^ *Jabuticaba (Myrciaria cauliflora)*, also known as Brazilian grape, is a hard-skinned berry of the *Myrtaceae* family, largely distributed in Brazil. It can be consumed fresh or in the form of liqueurs, wines, jams and sweets, and its consumption has increased in Brazil and worldwide.^8,9^ In addition to the use of *jabuticaba* as food and beverage, in folk medicine, that fruit is used to treat some diseases, such as asthma, inflammations, and gastrointestinal and cardiovascular disorders.^10^ Recent findings have shown that *jabuticaba* can decrease oxidative process,^11^ hyperglycemia associated with insulin resistance^12^ and dyslipidemia.^13^ In addition, that species has a proven endothelium-dependent hypotensive and vasodilating effect, mediated by nitric oxide pathway.^14^

Considering that *jabuticaba* has important biological effects and that its action on the cardiovascular system has been little studied, this study was aimed at assessing the possible effect of the *jabuticaba* extract directly on the VSM, mainly its effect on Ca^2+^ influx through the plasma membrane and activation of K^+^ channels.

## Methods

### Preparation of jabuticaba extract

For this study, the plant specimens were donated by the "Jabuticabal" wine house in the city of Hidrolândia, Goiás state, Brazil. A sample of the plant specimen was stored at the herbarium of the department of botany of the Federal University of Goiás (UFG), Goiânia, Goiás state, Brazil (n. 21140). Seedless fruits were dried in a greenhouse with air circulation, powdered in a pulverizer mill and passed through a 60-mesh sieve at the Laboratory of Research on Natural Products, Pharmacy School/UFG. The powder obtained was stored at -20°C. To prepare the extract, the dried material was exhaustively percolated into an ethanol:water solution (55:45 v/v), and the material obtained was filtered and submitted to rotary evaporation under reduced pressure at 40°C, resulting in the ethanol-free *jabuticaba* hydroalcoholic extract (JHE). After that process, the JHE was maintained in a freezer (-20°C) protected from light. On the days of experiment, the JHE was solubilized in distilled water at the concentration of 120 mg/mL.

The phytochemical characterization and pattern of the JHE showed 17.89% of phenolic compounds, quantified by using the Hagerman and Butler method, adapted by Mole and Watermen.^15^ The JHE showed ellagic acid (phytochemical marker, determined by HPLC-PDA) at 0.222% concentration.^14^ According to Abe et at.,^9^ the total ellagic acid content in *M. cauliflora* fruits ranges from 0.021% to 0.311%. Thus, that phytochemical marker concentration in JHE is in accordance with the fruit content.

### Animals and preparation of isolated arteries

Wistar male rats (200-230 g) from the central vivarium of the UFG were used in this study. All experimental protocols abided by the UFG Animal Research Ethics Committee (protocol: 015/2014). This study is in accordance with the European Union Guide to the Care and Use of Experimental Animals (2010/63/UE).

The rats were sacrificed by use of cervical dislocation under inhalation anesthesia. Thoracic aorta was isolated, cleared of connective and adipose tissues, and sliced into rings (± 4 mm), which were mounted between two metal hooks, one of which was connected to a power transducer to record isometric tension (DATAQ Instruments, Akron, OH, USA) and the other was fixed to a cube for the isolated organ. The rings were placed into chambers for isolated organs, containing modified Krebs solution [composition in mM: NaCl, 130.0; KCl, 4.7; KH_2_PO_4_, 1.2; CaCl_2_,1.6; MgSO_4_, 1.2; NaHCO_3_, 14.9; glucose, 5.5], at pH of 7.4, under gasification with carbogen mixture (95% O_2_ + 5% CO_2_) at 37°C, and maintained at baseline tension of 1 g (ideal resting tension, previously standardized at our laboratory). To prevent the influence of vascular-endothelium-derived factors, endothelial cells were mechanically removed by rubbing the vessel lumen with a thin metal rod, the effectiveness of the removal being evidenced by lack of relaxation due to acetylcholine (1 *µ*M) in aortic rings pre-contracted with EC_50_ of phenylephrine (0.1 *µ*M).

### Experimental protocols

After 60 minutes of stabilization at baseline tension (1 g), the arteries were pre-contracted with phenylephrine (0.1 *µ*M), and cumulative relaxation-concentration-effect curves were built for JHE (0 to 1.92 mg/mL) and for verapamil, used as inner control (10 nM to 100 *µ*M).

To assess the cellular pathways responsible for the relaxant effect of JHE, aortic rings were pre-contracted with phenylephrine (0.1 *µ*M) for 20 minutes after incubation with the following agents: 1) Ca^2+^ ATPase of the sarcoplasmic reticulum, cyclopiazonic acid (CPA, 10 *µ*M); 2) non-selective K^+^ channel blocker, tetraethyl-ammonium (TEA, 1 mM); 3) selective voltage-gated K^+^ channel (K_v_) blocker, 4-aminopyridine (4-AP, 1 mM); 4) selective ATP-sensitive K^+^ channel (K_ATP_) blocker, glibenclamide (3 *µ*M); 5) Ca^2+^-dependent K^+^ channel (K_Ca_) blocker, clotrimazole (5 *µ*M).

To assess the influence of JHE on the contraction induced by adrenergic contractile agonist, concentration-effect curves were built for phenylephrine (selective α1-adrenergic agonist, 0.1 nM to 10 *µ*M) in the presence (20 minutes) or absence of JHE at inhibitory concentration 50% (IC_50_, 0.5 mg/mL) or 100% (IC_100_, 1.92 mg/mL). In addition, the inhibitory effect of the Ca^2+^ channel blocker verapamil (IC_50_, 0.3 *µ*M) was assessed as inner control.

In another series of experiments, JHE action on Ca^2+^ influx stimulated by two different agents was analyzed. The preparations were initially contracted with a KCl solution (75 mM) to cause maximum contraction of each preparation (100% contraction), being then rinsed with Ca^2+^-free Krebs solution until total relaxation. To exhaust the intracellular storage of Ca^2+^, the preparations were stimulated to contract with phenylephrine in Ca^2+^-free Krebs solution until any contractile response disappeared (approximately 5 or 6 times, for 30-50 minutes). Then the preparations were rinsed several times with Ca^2+^-free Krebs solution, and then incubated for 20 minutes with JHE at inhibitory concentration 50% (IC_50_, 0.51 mg/mL) or 100% (IC_100_, 1.92 mg/mL). In addition, the inhibitory effect of the Ca^2+^ channel blocker verapamil (IC_50_, 0.3 *µ*M) was assessed as inner control. After incubation, the contractile stimulus was applied (phenylephrine, 0.1 *µ*M, or KCL, 75 mM), and concentration-effect curves were built for CaCl_2_ (0 to 3.0 mM).

### Statistical analysis

The results of isometric tension were expressed as mean ± standard error of the mean (SEM) of at least five experiments (n = 5-8) obtained from different animals. The graphs were built and analyzed by use of the GraphPad Prism software (GraphPad Software Corporation, 5.0 version) with ANOVA and Bonferroni post-test. The 5% significance level (p < 0.05) was adopted for the differences.

## Results

### Relaxant effect of JHE on isolated arteries

**Figure 1:**
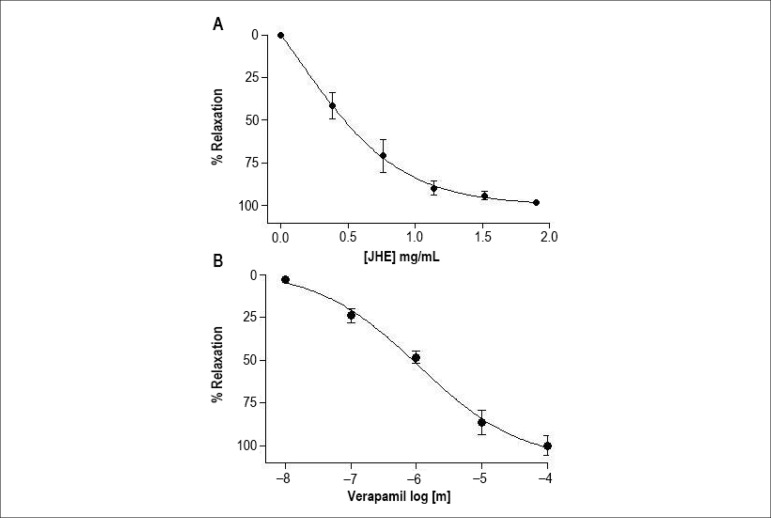
Cumulative concentration-response curves of jabuticaba hydroalcoholic extract (JHE) (A) and verapamil (B) in isolated endothelium-denuded arteries. The points represent mean ± SEM of the relaxant effect expressed as % relaxation.

The JHE caused relaxation in preparations of endothelium-denuded arteries on a concentration-dependent way, relaxation initiating at the concentration of 0.38 mg/mL, and achieving the maximum effect (E_max_) of 98.3% ± 0.4% (n = 6) at the concentration of 1.92 mg/mL (IC_100_) (Figure 1A). The JHE concentration that induced 50% relaxation (IC_50_) was 0.51 mg/mL. Similarly, verapamil (used as positive control) induced concentration-dependent relaxation with E_max_ of 99.8% ± 1.8% (n = 5) and IC_50_ of 0.3 *µ*M.

### Effect of JHE on the phenylephrine-induced contraction

**Figure 2:**
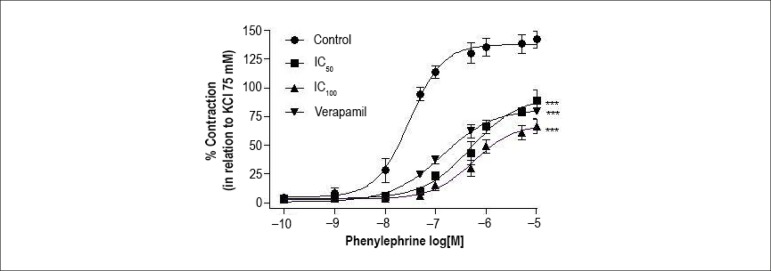
Effect of jabuticaba hydroalcoholic extract (JHE) and verapamil on the phenylephrine-induced contraction in isolated endothelium-denuded arteries. Cumulative concentration-response curves were built in control conditions and after incubation (20 min) with JHE (IC_50_: 0.51 or IC_100_: 1.92 mg/mL) or verapamil (IC_50_: 0.3 µM). The points represent mean ± SEM of the contractile effect expressed as % contraction in relation to total KCl-induced contraction (75 mM). Significant difference: *** p<0.001 vs. Control

The E_max_ value for phenylephrine (142.1% ± 7.1%, n = 6) was significantly (p < 0.001) reduced to 88.7% ± 6.2% (n = 5), 66.1% ± 5.1% (n = 6) and 79.9% ± 5.5% (n = 5) after incubation with IC_50_ and IC_100_ of JHE or verapamil, respectively. The addition of IC_50_ and IC_100_ of JHE or verapamil significantly increased phenylephrine pD_2_ values (-log EC_50_) from 6.24 ± 0.09 to 5.35 ± 0.04, 5.14 ± 0.09 and 5.68 ± 0.07, respectively (Figure 2).

### Effect of JHE on Ca^2+^-influx-induced contraction in preparations stimulated with phenylephrine or KCl

**Figure 3:**
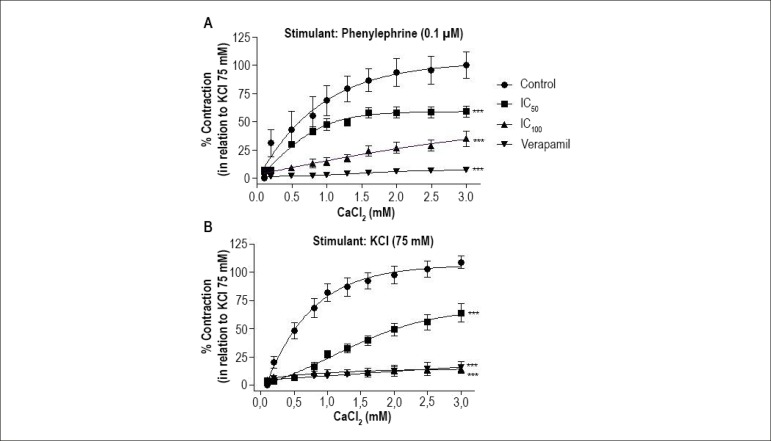
Effect of jabuticaba hydroalcoholic extract (JHE) and verapamil on the contractile response in isolated endothelium-denuded arteries. Cumulative concentration-response curves for CaCl_2_ were stimulated with phenylephrine (0.1µM) (A) or KCl 75 mM (B) in control conditions and after incubation (20 min) with JHE (IC_50_: 0.51 or IC_100_: 1.92 mg/mL) or verapamil (IC_50_: 0.3 µM). The points represent mean ± SEM of the contractile effect expressed as % contraction in relation to total KCl-induced contraction (75 mM). Significant difference: *** p<0.001 vs. Control.

Regarding the Ca^2+^-influx-induced contraction stimulated by phenylephrine, pre-incubation with JHE (IC_50_ or IC_100_) significantly reduced (p < 0.001) the E_max_ values from 106.8% ± 7.5% (n = 5) to 58.8% ± 4.9% (n = 6) and 34.5% ± 3.2% (n = 6), respectively. In addition, treatment with verapamil significantly reduced (p < 0.001) the contraction to 7.1% ± 1.1% (n = 5) (Figure 3A).

Regarding the Ca^2+^-influx-induced contraction stimulated by KCl (75 mM), pre-incubation with JHE (IC_50_ or IC_100_) significantly reduced (p < 0.001) the E_max_ values from 108.8% ± 4.3% (n = 5) to 63.8% ± 6.1% (n = 6) and 14.6% ± 1.9% (n=6), respectively. In addition, treatment with verapamil significantly reduced (p < 0.001) the contraction to 15.5% ± 1.1% (n=6) (Figure 3B).

### Effect of reticular Ca^2+^ ATPase inhibitor, CPA, and K^+^-channel blockers on JHE- induced relaxation

**Figure 4:**
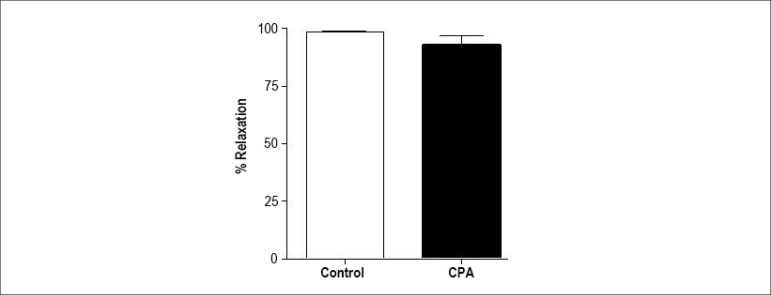
Maximum relaxant effect induced by jabuticaba hydroalcoholic extract (JHE) in isolated arteries pre-contracted with phenylephrine (0.1 µM) in the absence or presence (20 min) of the reticular Ca^2+^ ATPase inhibitor, cyclopiazonic acid (CPA, 10 µM). The bars represent mean ± SEM of the maximum relaxant effect expressed as % relaxation.

Treatment with CPA did not change the JHE-induced relaxation (93.8% ± 4.6%, n = 6) in isolated arteries (Figure 4). Thus, JHE did not change the inner Ca^2+^ uptake by the sarcoplasmic reticulum to induce vascular relaxation.

**Figure 5:**
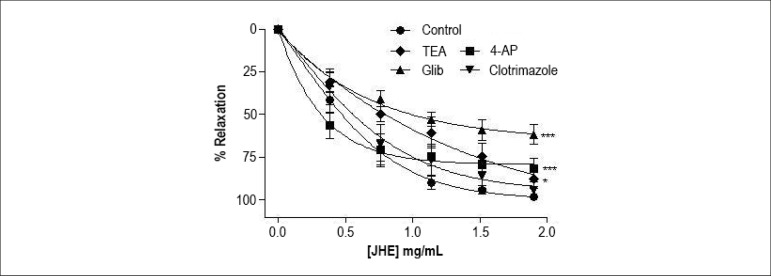
Effects of K+ channel blockers on the relaxation induced by jabuticaba hydroalcoholic extract (JHE) in isolated arteries pre-contracted with phenylephrine (0.1 µM) in the absence or presence (20 min) of the blockers tetraethyl-ammonium (TEA, 1 mM), glibenclamide (Glib, 3 µM), clotrimazole (5 µM) and 4-aminopyridine (4-AP, 1 mM). The points represent mean ± SEM of the relaxant effect expressed as % relaxation. Significant difference: *p < 0.05; *** p < 0.001 vs. Control

As shown in figure 5, except for clotrimazole (94.1% ± 4.5%, n = 5), K^+^-channel blockers changed the JHE-stimulated relaxation. The JHE-induced relaxation (E_max_: 98.3% ± 0.4%, n = 6) was significantly (p < 0.05) reduced by TEA (E_max_: 87.6% ± 5.7%, n=5), glibenclamide (E_max_: 61.6% ± 5.8%, n = 6) and 4-AP (E_max_: 81.6% ± 5.9%, n = 5). The results showed that JHE-induced relaxation depends on K^+^ efflux through the membrane.

## Discussion

The major finding of this study is that JHE, in addition to having a hypotensive effect and inducing vascular relaxation through endothelial nitric oxide pathway, as shown by our team,^14^ acts directly on VSM and leads to endothelium-independent relaxation. Therefore, *jabuticaba* clearly has cardiovascular effects through multiple endothelium-dependent and independent pathways. It is worth noting that the JHE concentration capable of inducing 100% vascular relaxation through the endothelial pathway is approximately 16 times lower (0.12 mg/mL)^14^ than the JHE concentration necessary to induce 100% relaxation acting directly on VSM (1.92 mg/mL).

Blood vessel contraction and relaxation in response to physiological demands are controlled by changes in intracellular Ca^2+^ concentration of VSM. The Ca^2+^ used for contraction includes intracellular or extracellular sources, or both. Sarcoplasmic reticulum is the major source of intracellular Ca^2+^.^16^ Our experiments showed that JHE does not change Ca^2+^ uptake by the sarcoplasmic reticulum, because its selective inhibitor, CPA, did not change the relaxation profile.

Voltage-gated Ca^2+^ channels (VGCC), also known as L-type Ca^2+^ channels, and receptor-operated Ca^2+^ channels (ROCC) located on the plasma membrane of VSM cells play a fundamental role in controlling Ca^2+^ influx.^17,18^ Phenylephrine-induced contraction is mediated by Ca^2+^ influx increase via VGCC and ROCC.^19,20^ However, contraction induced by membrane depolarization, such as in high KCl concentrations, activates preferentially VGCC.^21^ The results of the present study show that treating arteries with JHE inhibits the vascular contraction induced by the adrenergic stimulus with phenylephrine, suggesting that JHE blocks Ca^2+^ influx by interfering with VGCC and/or ROCC.

In an attempt to clarify the cell mechanism through which JHE induces vascular relaxation, experiments were performed in a Ca^2+^-free solution. Two different stimuli, phenylephrine and KCl (75 mM), were used to induce Ca^2+^ influx. The JHE, as well as verapamil, used as a positive control, inhibited the Ca^2+^-influx-induced contraction mediated by both stimuli. Because membrane depolarization with high concentrations of K^+^ activates specifically VGCC, we suggest that JHE acts directly or indirectly by blocking Ca^2+^ influx through the plasma membrane, acting preferentially on VGCC.

Natural products have constantly shown the involvement of K^+^ channels in their vasodilating mechanism.^22^ Several types of K^+^ channels, such as ATP-sensitive K^+^ channels (K_ATP_), Ca^2+^-dependent K^+^ channels (K_ca_) and voltage-gated K^+^ channels (K_v_), are present in VSM.^23,24^ Those channels can be blocked by glibenclamide, clotrimazole and 4-AP, respectively.^24,25^ Tetraethyl-ammonium is a non-selective blocker of those channels. When activated, those channels allow K^+^ efflux, hyperpolarizing the VSM plasma membrane. This reduces Ca^2+^ influx through the VGCC and induces vasodilation.^26,27^ The present study shows that JHE-induced relaxation in endothelium-denuded arteries is hindered after K^+^ channel blockade. Except for clotrimazole, the other blockers hindered vascular relaxation, allowing relating its activation to the JHE effect.

Our results point to a new biological effect of *jabuticaba*, a Brazilian native specimen that has important biological effects on the cardiovascular system, such as glucose-lowering,^12^ lipid-lowering^13^ and hypotensive effects.^14^ Thus, the biological *jabuticaba*-induced effects demonstrated in this study will contribute to increase the knowledge about *jabuticaba*-derived compounds and their use as medicinal plant or functional food to prevent cardiovascular problems.

## Conclusion

This study shows that JHE induces endothelium-independent vasodilation. The major cellular pathways used by JHE to cause vascular relaxation are inhibition of the Ca^2+^-influx through plasma membrane, in addition to K^+^ channel activation in VSM cells.
